# Fine-Mapping and Initial Characterization of QT Interval Loci in African Americans

**DOI:** 10.1371/journal.pgen.1002870

**Published:** 2012-08-09

**Authors:** Christy L. Avery, Praveen Sethupathy, Steven Buyske, Qianchuan He, Dan-Yu Lin, Dan E. Arking, Cara L. Carty, David Duggan, Megan D. Fesinmeyer, Lucia A. Hindorff, Janina M. Jeff, Liviu Klein, Kristen K. Patton, Ulrike Peters, Ralph V. Shohet, Nona Sotoodehnia, Alicia M. Young, Charles Kooperberg, Christopher A. Haiman, Karen L. Mohlke, Eric A. Whitsel, Kari E. North

**Affiliations:** 1 Department of Epidemiology, University of North Carolina at Chapel Hill, Chapel Hill, North Carolina, United States of America; 2 Department of Genetics, University of North Carolina at Chapel Hill, Chapel Hill, North Carolina, United States of America; 3 Carolina Center for Genome Sciences, University of North Carolina at Chapel Hill, Chapel Hill, North Carolina, United States of America; 4 Department of Statistics and Biostatistics, Rutgers University, Piscataway, New Jersey, United States of America; 5 Department of Genetics, Rutgers University, Piscataway, New Jersey, United States of America; 6 Department of Biostatistics, University of North Carolina at Chapel Hill, Chapel Hill, North Carolina, United States of America; 7 McKusick-Nathans Institute of Genetic Medicine and Division of Cardiology, Department of Medicine, Johns Hopkins University School of Medicine, Baltimore, Maryland, United States of America; 8 Fred Hutchinson Cancer Research Center, Seattle, Washington, United States of America; 9 Translational Genomics Research Institute, Phoenix, Arizona, United States of America; 10 Office of Population Genomics, National Human Genome Research Institute, National Institutes of Health, Bethesda, Maryland, United States of America; 11 Department of Medicine, Vanderbilt University, Nashville, Tennessee, United States of America; 12 Department of Medicine, University of California San Francisco, San Francisco, California, United States of America; 13 Division of Cardiology, University of Washington, Seattle, Washington, United States of America; 14 Center for Cardiovascular Research, John A. Burns School of Medicine, University of Hawaii, Honolulu, Hawaii, United States of America; 15 Department of Preventive Medicine, Keck School of Medicine and Norris Comprehensive Cancer Center, University of Southern California, Pasadena, California, United States of America; 16 Department of Medicine, University of North Carolina at Chapel Hill, Chapel Hill, North Carolina, United States of America; University of California San Diego and The Scripps Research Institute, United States of America

## Abstract

The QT interval (QT) is heritable and its prolongation is a risk factor for ventricular tachyarrhythmias and sudden death. Most genetic studies of QT have examined European ancestral populations; however, the increased genetic diversity in African Americans provides opportunities to narrow association signals and identify population-specific variants. We therefore evaluated 6,670 SNPs spanning eleven previously identified QT loci in 8,644 African American participants from two Population Architecture using Genomics and Epidemiology (PAGE) studies: the Atherosclerosis Risk in Communities study and Women's Health Initiative Clinical Trial. Of the fifteen known independent QT variants at the eleven previously identified loci, six were significantly associated with QT in African American populations (*P*≤1.20×10^−4^): *ATP1B1*, *PLN1*, *KCNQ1*, *NDRG4*, and two *NOS1AP* independent signals. We also identified three population-specific signals significantly associated with QT in African Americans (*P*≤1.37×10^−5^): one at *NOS1AP* and two at *ATP1B1*. Linkage disequilibrium (LD) patterns in African Americans assisted in narrowing the region likely to contain the functional variants for several loci. For example, African American LD patterns showed that 0 SNPs were in LD with *NOS1AP* signal rs12143842, compared with European LD patterns that indicated 87 SNPs, which spanned 114.2 Kb, were in LD with rs12143842. Finally, bioinformatic-based characterization of the nine African American signals pointed to functional candidates located exclusively within non-coding regions, including predicted binding sites for transcription factors such as TBX5, which has been implicated in cardiac structure and conductance. In this detailed evaluation of QT loci, we identified several African Americans SNPs that better define the association with QT and successfully narrowed intervals surrounding established loci. These results demonstrate that the same loci influence variation in QT across multiple populations, that novel signals exist in African Americans, and that the SNPs identified as strong candidates for functional evaluation implicate gene regulatory dysfunction in QT prolongation.

## Author Summary

The QT interval (QT) provides a measure of a ventricular action potential, and its prolongation is associated with sudden death and ventricular arrhythmias. Genome-wide association studies performed in European populations have identified common genetic variants that influence QT. However, it is unclear whether these variants are relevant in other populations, including African Americans. The increased genetic diversity in African Americans also provides opportunities to narrow association signals and identify candidates for functional evaluation. We therefore used data from 8,644 African Americans to further characterize previously identified QT loci. Of the fifteen known independent QT variants at the eleven previously identified QT loci, six were associated with QT in African Americans. We also identified three variants that were independent from previously reported signals and narrowed intervals flanking association signals using patterns of linkage disequilibrium. Finally, bioinformatic-based characterization pointed to candidates located outside protein coding regions. Our results underscore the utility of genetic studies in African ancestral populations to identify novel variants and narrow intervals surrounding established loci. These results suggest that known QT loci are important in African Americans and that further characterization of these loci in other populations may provide additional insights into the genetic and molecular mechanisms underlying QT.

## Introduction

The QT interval (QT), as measured by the resting 12-lead electrocardiogram (ECG), reflects the duration of ventricular depolarization and repolarization, providing a non-invasive assessment of an average ventricular action potential. QT prolongation is an established risk factor for ventricular tachyarrhythmias [1], coronary heart disease [2], and sudden cardiovascular as well as all-cause death [3]. Although numerous factors influencing QT have been identified, including heart rate [4], structural heart disease [5], [6], gender [7], [8], age [9], and medication use [10]–[12], a large portion of the variance in QT remains unexplained.

Several lines of evidence support a genetic contribution to QT. Initial evidence was provided by studies of inherited cardiac arrhythmias including long- and short-QT syndromes, which identified rare and highly penetrant mutations in ion channel and ion channel associated genes associated with QT [13]. Family studies have also reported that ventricular repolarization (as measured by QT) is heritable [14]–[17]. In addition, recent genome-wide association (GWA) studies performed in populations of predominantly European descent have identified common SNPs in twelve loci, including *NOS1AP*, *KCNH2*, and *PLN* [18]–[23], that influence the distribution of QT.

To date, the majority of published GWA studies examining QT have been performed in populations of European descent, although one study also examined an Indian Asian population [23]. It is therefore unclear whether previously identified QT loci are relevant in other racial groups such as African Americans or whether there are population-specific SNPs influencing QT. Furthermore, the increased genetic diversity in populations of African ancestry provides opportunities for the narrowing and fine-mapping of loci identified in European and Indian Asian populations [24]. Fine-mapping, which includes the dense genotyping of common and rare SNPs at already established loci, is a helpful next step in the identification of functional polymorphisms underlying the QT distribution. For example, dense genotyping can capture rarer SNPs that may be inadequately represented on frequently used genome-wide genotyping arrays, and which may have large effects, thereby potentially helping to explain a larger fraction of the QT heritability [25], which is estimated to range from 35% to 52% [14]–[17].

In this study, we evaluated eleven QT loci previously identified in populations of European and Indian Asian descent in 8,644 African American participants from the Atherosclerosis Risk in Communities study (ARIC) and Women's Health Initiative clinical trial (CT). In addition to testing the previously reported QT index SNPs at the known loci, we also searched for stronger markers of the index signal and investigated evidence for independent, novel SNPs influencing QT in African Americans. For loci associated with QT in African Americans, we also investigated whether patterns of linkage disequilibrium (LD) within African Americans could narrow the regions likely to harbor the biologically relevant variant. Finally, we queried bioinformatic databases and performed related *in silico* analyses to identify potential candidate polymorphisms for follow-up functional evaluation.

## Results

Participants are drawn from two separate studies (Methods), which together comprise 9,702 individuals. After applying the exclusion criteria (Methods), phenotypic data from 8,644 participants are included in this analysis. The majority of participants are female (86%) and ages range from 45–79 years (Table S1). Estimates of mean QT and heart rate are consistent across the studies. Approximately 60% of participants are directly genotyped on the Metabochip; the remaining 40% have genotypes imputed from the Affymetrix 6.0 panel (Methods).

### Representation of QT loci on the Metabochip and imputation

The Metabochip, a custom array containing approximately 195,000 SNPs, is designed to facilitate fine-mapping of loci associated with cardiovascular and metabolic traits, including QT, blood pressure, cholesterol, type 2 diabetes, and anthropometrics. To identify and fine-map signals associated with QT in African Americans, 6,670 SNPs from eleven previously identified QT loci represented on the Metabochip are examined (Table S2; Methods). The number of SNPs at each locus with minor allele frequency (MAF) estimates ≥1% ranges from 51 to 1,371, corresponding to regions spanning 67 Kb to 664 Kb in size (median size: 275 Kb).

On average, imputation quality for metabochip SNPs imputed in WHI SNP Health Association Resource (SHARe) participants (n = 3,531) is high across the 11 loci, with the exception of *RNF207*, *KCNH2*, and *KCNQ1* (Table S2). As described in the Methods, SNPs with imputation quality scores <0.95 are discarded.

### Association of QT loci in African Americans

The six published GWA studies of QT (five studies of European ancestral populations, one study of European and Indian Asian ancestral populations) reported 25 index SNPs (*P*≤5.0×10^−8^, Table 1; D' estimates provided in Table S3) across eleven loci, which together represent fifteen independent signals (at r^2^≥0.20 in European ancestry populations). The fifteen signals include three independent signals at *NOS1AP* and two independent signals at both *PLN* and *KCNH2*.

**Table 1:** Associations with common variants at fifteen previously reported QT loci across eleven chromosomes in n = 8,644 African American participants.

Index SNPs from Published GWA studies in European and Indian Asian populations								Best marker in African Americans a					r^2^ with index SNP	
Locus	Position	Ind. signal	Index SNP	Alleles	CAF		*P*-value (AF)	Marker	BP (build 36)	Alleles	CAF	*P*-value	EU^b^	AF^c^
					EU^b^	AF^c^								
*RNF207*	1p36.31	1	rs846111 [20], [22]	C/G	0.29	0.056	0.22	rs7525357	6223091	C/G	0.56	0.010	—	—
*NOS1AP*	1q23.3	1	rs12143842 [20]–[22]	T/C	0.24	0.13	9.0×10^−11^	rs12143842	160300514	T/C	0.13	9.0×10^−11^	1.0	1.0
			rs2880058 [19], [23]	A/G	0.30	0.29	2.4×10^−4^	—	—	—	—	—	0.69	0.052
			rs10494366^d^ [18]	A/G	0.64	0.60	0.029	—	—	—	—	—	0.53	0.026
		2	rs12029454 [20]	A/G	0.15	0.28	5.5×10^−6^	rs72633699	160431793	T/C	0.30	6.1×10^−7^	0.92	0.079
			rs4657178 [22]	T/C	0.27	0.36	0.92	—	—	—	—	—	0.46	0.007
		3	rs16857031 [20]	G/C	0.12	0.29	7.2×10^−3^	rs76204833	160364953	A/C	0.95	7.0×10^−4^	—	—
*ATP1B1*	1q24.2	1	rs10919071 [22]	A/G	0.89	0.97	3.0×10^−4^	rs10919062	167355571	T/C	0.97	6.7×10^−5^	0.94	0.98
*SCN5A*	3p22.2	1	rs12053903 [20]	C/T	0.33	0.81	0.70	rs7374391	38573339	T/C	0.08	0.11	—	—
			rs11129795 [22]	A/G	0.26	0.18	0.20	—	—	—	—	—	—	—
*PLN*	6q22.31	1	rs11970286 [22]	T/C	0.49	0.22	0.10	rs56403768	118810227	T/C	0.77	3.8×10^−5^	0.88	0.48
			rs11153730 [21]	T/C	0.53	0.71	0.034	—	—	—	—	—	0.76	0.50
			rs11756438 [20]	A/C	0.48	0.36	0.60	—	—	—	—	—	0.68	0.39
		2	rs12210810 [22]	C/G	0.037	0.010	0.70	rs35407905	118996921	A/G	0.05	0.029	—	—
*KCNH2*	7q36.1	1	rs2968863 [22]	T/C	0.22	0.053	0.074	rs56282717	150288028	A/G	0.05	0.032	—	—
			rs2968864^e^ [20]	NA	NA	NA	NA	—	—	—	—	—	—	—
		2	rs4725982 [20]	T/C	0.20	0.26	0.010	rs1805120	150280464	A/G	0.24	0.0045	—	—
*KCNQ1*	11p15.5	1	rs12296050 [22]	T/C	0.81	0.52	8.6×10^−5^	rs12296050	2445918	T/C	0.52	8.6×10^−5^	1.0	1.0
			rs12576239 [20]	T/C	0.13	0.18	0.48	—	—	—	—	—	0.55	0.12
			rs2074238^f^ [20]	NA	NA	NA	NA	—	—	—	—	—	—	—
*LITAF*	16p13.13	1	rs8049607 [20], [22]	T/C	0.49	0.45	0.057	rs735951	11601037	A/G	0.44	0.023	—	—
*NDRG4*	16q21	1	rs7188697^d^ [22]	T/C	0.75	0.85	6.2×10^−5^	rs7184114	57142870	A/C	0.83	4.4×10^−5^	0.76	0.90
			rs37062^d^ [20]	A/G	0.24	0.16	8.0×10^−5^	—	—	—	—	—	0.74	0.92
*LIG3*	17q12	1	rs2074518 [20]	T/C	0.49	0.22	0.13	rs3744358	30361027	T/G	0.83	0.076	—	—
*KCNJ2*	17q24	1	rs17779747 [22]	T/G	0.32	0.097	0.62	rs12103757	65986428	A/G	0.51	0.022	—	—

a Restricted to SNPs with minor allele frequency ≥0.01.

b Calculated in the Malmö Diet and Cancer Study or 1,000 Genomes CEU data when Malmö data unavailable.

c Calculated in the Atherosclerosis Risk in Communities Study.

d SNP not present on Metabochip, SNP proxy substituted.

e SNP not present on Metabochip, but in very high LD with rs2968863 (r^2^>0.95).

f SNP failed quality control and no proxy was available.

AF, African American. BP, base pair. CAF, coded allele frequency. Est, estimate. EU, European. GWA, genome wide association. Ind, independent. NA, not available. SE, standard error. SNP, single nucleotide polymorphism.

We first test the fifteen independent signals for association with QT in African Americans. Specifically, we evaluate the index SNPs for each of the fifteen independent signals as well as all SNPs in LD with the index SNPs (r^2^≥0.20 using European ancestral LD patterns) to determine whether any of the fifteen independent signals generalize to African Americans. The significance criterion, α_a_ = 1.20×10^−4^, is based on the number of tag SNPs in African Americans that capture (r^2^≥0.80, using African American LD patterns) all SNPs that are correlated with the index SNPs (r^2^≥0.20; determined using European ancestral LD patterns; see Methods).

Six of the fifteen independent signals are significantly associated with QT in African Americans (Table 1): *NOS1AP* independent signals 1 and 2, *ATP1B1*, *PLN* independent signal 1, *KCNQ1*, and *NDRG4*. Of those that are not significantly associated with QT (*P*-value>1.20×10^−4^), estimates for index SNPs representing the two *KCNH2* independent signals as well as the *RNF207* index SNP show a consistent magnitude and direction of effect when compared to published estimates (Table S4).

The best marker in African Americans for the first *NOS1AP* independent signal (rs12143842) and *KCNQ1* (rs12296050) is identical to the European index SNP. For the *ATP1B1* and *NDRG4* index SNPs as well as the second *NOS1AP* independent signal, the best marker in African Americans shows only a slightly more significant association than the index signal (<1 order of magnitude change in the *P*-value; Table 1). In contrast, at the first *PLN* independent signal, the three index SNPs are not significantly associated with QT in African Americans (*P*-value range: 0.034–0.60), although a substantially stronger marker of the index signal is detected (rs56403768; *P*-value = 3.8×10^−5^). In Europeans, rs56403768 is correlated with the three index SNPs (LD r^2^ range: 0.68–0.88). However, patterns of correlation are weaker in African Americans (LD r^2^ range: 0.39–0.50).

**Figure 1:**
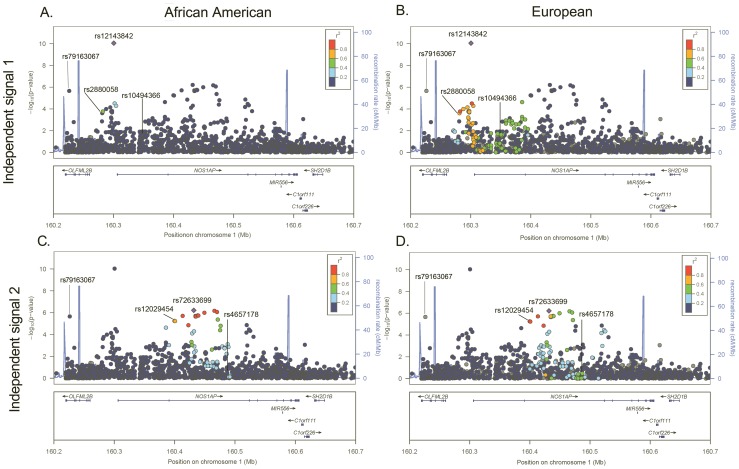
−Log *P* plot for common SNPs at the *NOS1AP* independent signal 1 and 2 loci. *P*-values are estimated in African Americans and are plotted using linkage disequilibrium estimates from African Americans (panels A and C) and Europeans (panels B and D). SNPs are represented by *circles*, lines indicate index SNPS previously identified in GWA studies of European and Indian Asian populations, and the *large blue diamond* is the best marker in African Americans. Circle color represents correlation with the best marker in African Americans: *blue* indicates weak correlation and *red* indicates strong correlation. Recombination rate is plotted in the background and annotated genes are shown at the bottom of the plot.

We then evaluate evidence for additional independent signals at the eleven previously identified QT loci, focusing on SNPs that are uncorrelated with the index signals in European populations. Here, statistical significance is defined using an efficient Monte Carlo approach that accounts for LD between SNPs at the eleven previously identified QT loci (α_b_ = 1.37×10^−5^; see Methods). Eleven SNPs at two loci that are uncorrelated with the index SNPs (r^2^≤0.20; evaluated using both African American and European ancestral LD patterns) exceed our significance threshold. Conditional analysis confirms that these eleven SNPs represent three novel associations, one flanking the *NOS1AP* locus and two residing within or nearby *ATP1B1* (Table 2; Figures 1 and S1). Of note, the novel *NOS1AP* SNP is monomorphic in populations of European ancestry. Together, the six best markers in African Americans and the three novel SNPs explain 1.6% of the variance in heart rate-corrected QT.

**Table 2:** Novel and independent SNPs associated with QT at two previously identified QT loci in n = 8,644 African American participants.

African American index SNP^a^	Locus	Chr	Position (Build 36)	Alleles^b^	Coded allele frequency		*P*-value
					African Americans^c^	Europeans^d^	
rs79163067	*NOS1AP*	1	160226234	A/G	0.016	0	2.2×10^−6^
rs12061601	*ATP1B1*	1	167337074	T/C	0.75	0.86	5.6×10^−6^
rs10919095^e^	*ATP1B1*	1	167447481	T/C	0.25	0.48	6.9×10^−6^

a Restricted to SNPs with minor allele frequency ≥0.01 that passed quality control and defined as locus-specific SNP with the lowest *P*-value.

b Coded allele listed first.

c Calculated in the Atherosclerosis Risk in Communities Study.

d Calculated in the Malmö Diet and Cancer Study.

e Adjusted for rs12061601.

Est, estimate. SE, standard error. SNP, single nucleotide polymorphism.

Next, we examine whether African American LD patterns can assist with the narrowing of association signals at the six loci that generalized to African Americans. An example of variation in LD patterns by ancestral population is shown by the first *NOS1AP* independent signal. Among African Americans, 0 SNPs are correlated (r^2^≥0.50) with rs12143842, which is the best marker in African Americans and the index SNP reported by three prior QT GWA studies (Figure 1; Table 3) [20]–[22]. This is in contrast to LD patterns estimated in Europeans for *NOS1AP* independent signal 1, where 87 SNPs are correlated with the three index signals that characterize this independent signal (r^2^≥0.50), representing a region spanning 114.2 Kb. For the five remaining markers (Figures 1 and S1, S2, S3, S4), African American populations exhibit lower levels of LD as compared to European populations (mean narrowing = 48.2 Kb). Likewise, fine-mapping in African Americans helped to narrow the association signals for all six loci.

**Table 3:** Comparison of linkage disequilibrium patterns between populations of African and European descent for six previously identified QT loci significantly associated with QT in n = 8,644 African American participants.

		African Americans		Europeans		
Locus	Ind. signal	N. SNPs in LD with best marker^a^^,^ b	Region size (Kb)	N. SNPs in LD with index SNPs^a^^,^ c	Region size (Kb)	Region size difference (Kb)^d^
*NOS1AP*	1	0	0	87	114.2	114.2
	2	13	71.8	29	86.4	14.6
*ATP1B1*	1	24	331.6	28	340.1	8.5
*PLN, SLC35F1*	1	27	343.7	173	466.0	122.3
*KCNQ1*	1	7	10.4	19	34.6	24.2
*NDRG4*	1	57	141.8	65	147.0	5.2

a r^2^≥0.50.

b Calculated using African American LD patterns.

c Calculated using European LD patterns.

d Calculated as (European region size - African American region size (Kb)).

Kb, kilobase. LD, linkage disequilibrium.

### Bioinformatic-based functional characterization of QT loci

Bioinformatic analysis of the six best markers in African Americans and the three novel and independent SNPs (Table S5; Methods) does not identify any correlated non-synonymous coding variants; instead, all signals harbor variants that occur solely within non-coding regions with the potential for influencing *cis*-regulation (Table S6). Several variants occur within candidate regulatory elements (promoter regions and DNase hypersensitive sites in human cardiomyocytes), including three (rs3864884, rs1646010, and rs27097) that are predicted to have allele-specific binding affinities for various transcription factors of relevance to cardiac function (Table S7). Though further functional characterization is outside the scope of this study, rs3864884, rs1646010, rs27097, and rs37036 represent compelling candidates for follow-up evaluation.

## Discussion

In this study composed of approximately 8,600 African American participants, we evaluated fifteen independent signals across eleven loci that were previously associated with QT in populations of European and Indian Asian ancestry at genome-wide significant levels. For five independent signals – the two *NOS1AP* independent signals, *ATP1B1*, *NDRG4*, and *KCNQ1* – the best markers in African Americans were either identical to or only slightly more significant than the index signal. These five markers are therefore not considered better signals than the index SNP. However, for the first *PLN* locus, the three previously identified index SNPs were not significantly associated with QT in African Americans. This result suggests that rs56403768, the best marker in African Americans, is a better proxy of a biologically important *PLN* allele and may help improve localization of the true association.

In addition to generalizing six previously characterized QT loci, we also identified three novel and independent signals for *NOS1AP* and *ATP1B1*. Notably, rs79163067, the novel *NOS1AP* signal, was monomorphic in European populations. When these three novel and independent variants were combined with the fifteen independent QT loci previously identified in populations of European and Indian Asian ancestry, our results suggest that to-date at least 18 independent variants influence QT.

Finally, we showed that evaluating LD patterns in admixed populations such as African Americans assisted with the narrowing of intervals flanking the putative causal variants. This narrowing was particularly evident for the first *NOS1AP* locus that included index SNP rs12143842, the most frequently reported SNP in the QT GWA study literature to-date. Rs12143842 also was the SNP that explained the majority of variance in heart rate-corrected QT as well as the best marker in African Americans for the first *NOS1AP* independent signal. Rs12143842 has yet to be evaluated in any functional studies, and although our bioinformatics characterization did not identify any compelling functional candidates, the SNP resides less than five Kb from the annotated *NOS1AP* promoter. Future studies evaluating the functional relevance of rs12143842 are clearly indicated.

Of the nine QT SNPs we identified (i.e. the six best markers in African Americans and the three novel SNPs), all resided in or were in LD with SNPs residing in candidate long-range regulatory elements in human cardiomyocytes, annotated promoter regions, and highly conserved non-coding elements, and as such, strongly implicate gene regulatory dysfunction in QT prolongation. One of the more striking predictions was with rs3864884, where the major allele [C] is predicted to bind an entirely different set of transcription factors (TFs) than the minor allele [T]. Notably, only the major allele is predicted to bind Hairy-related TFs, which are involved in regulating cardiac morphogenesis [26]. The minor allele is predicted to bind TBX5 and AhR; the former has been linked to a number of cardiac phenotypes including Holt-Oram syndrome [27] and atrioventricular conduction [28] and the latter regulates cardiac size [29], a known risk factor for QT-prolongation and cardiac sudden death [30]. Consistent with this prediction, the minor allele is associated with an increased QT in this population.

We were unable to identify associations with *RNF207*, *SCN5A*, *KCNH2*, *LITAF*, *LIG3*, and *KCNJ2* based on our statistical significance threshold. *RNF207* was reported in two previous QT GWA studies. However, because of design priorities, only 51 SNPs with MAF≥1% were available for analysis. Future fine-mapping efforts in African Americans or other admixed populations that include denser genotyping of this locus may therefore be useful.

*SCN5A*, *KCNH2*, and *LITAF* were all reported by two prior GWA studies of QT. *SCN5A* also has also been implicated in GWA studies of PR in populations of European [31] and African ancestry [28] as well as QRS interval duration in populations of European descent [32]. Our inability to detect signals at these three loci may simply reflect inadequate power, especially for *KCNH2*, for which estimates of the index SNPs in African Americans were consistent with the published literature. *KCNJ2* is a biologically plausible locus influencing QT, as it harbors mutations causing rare, familial forms of long QT syndrome [33]. Yet, the high *P*-values and the dense genotyping coverage of SNPs suggest that *KCNJ2* does not influence QT in African American populations.

The genetic architecture of African Americans and other admixed populations is on average characterized by lower correlation between SNPs when compared to European populations. Such populations therefore are valuable for the fine-mapping of previously identified loci, as fewer SNPs are expected to be correlated with the underlying functional variant, which is expected to be the same in populations of different ethnicity. We therefore anticipate that future fine-mapping efforts that include populations with different ethnic backgrounds will be useful for the further refinement of loci influencing QT as well as the identification of population-specific variants, as demonstrated by the current report.

Several limitations of the present study warrant further consideration in order to inform future efforts for fine-mapping and functional characterization of QT loci. First, although the Metabochip includes dense genotyping of most QT loci, it is possible that the causative variants are not included on the Metabochip. Second, our functional characterization is based on *in silico* analyses and requires experimental validation. Third, the majority of study participants were female. It is unclear how a predominantly female population may have influenced the results presented herein, considering the well-known dependence of QT on gender [7], [8]. Finally, our results, which are consistent with prior studies [22], show that common SNPs only explain a very small fraction of the variance in QT, although heritability estimates suggest a substantial genetic component. These modest effect sizes corroborate the multifactorial etiology of QT and demonstrate that substantially greater efforts are required to explain the “missing heritability”. Future efforts with increased sample sizes that examine rare variants, gene-gene and gene-environment interactions, and structural variants poorly captured on existing arrays are clearly needed [34].

In conclusion, our findings provide compelling evidence that the same genes influence variation in QT across ancestral populations and that additional, independent signals exist in African Americans. Moreover, all SNPs identified as strong candidates for functional evaluation implicate gene regulatory dysfunction. Further characterization of these loci, including direct sequencing and large-scale genotyping in African Americans and other admixed populations, may provide more information on the genetic and molecular mechanisms underlying QT.

## Materials and Methods

### Ethics statement

The Institutional Review Board at all participating institutions approved the study protocol. This study was conducted according to the principles expressed in the Declaration of Helsinki.

### Study populations

The Population Architecture using Genomics and Epidemiology (PAGE) study is a National Human Genome Research Institute funded effort examining the epidemiologic architecture of common genetic variants that have been reproducibly associated with human diseases and traits [35]. The PAGE study consists of a coordinating center and four consortia with access to large, diverse population-based studies including three National Health and Nutrition Examination Surveys, the Multiethnic Cohort, the WHI, the ARIC study, the Coronary Artery Risk Disease in Young Adults study, the Cardiovascular Health Study, the Hispanic Community Health Study/Study of Latinos, the Strong Heart Study, and the Strong Heart Family Study.

This PAGE Metabochip study included African American participants from the ARIC and WHI CT studies. Participants from the other PAGE studies were excluded from this effort due to the unavailability of ECG measures and/or genotype data. Genotypes of WHI CT participants were obtained in three phases: two sets of women were directly genotyped on the Metabochip platform by PAGE investigators during wave 1 (n = 797) and wave 2 (n = 1,128) and women (n = 3,531) with Metabochip variants imputed from previous genome-wide SNP data provided by the WHI SHARe [36]. Participants meeting the following criteria were excluded from the study: QT unavailable, atrial fibrillation/atrial flutter on ECG, left or right bundle branch block on ECG, QRS duration >120 milliseconds, intraventricular conduction delay on ECG, pacemaker implant antedating ECG, ancestry outlier, excessive heterozygosity, low call rate, or second member of first degree relative pair. Further details on the ARIC and WHI CT studies are provided in Text S1 (Participating Studies).

### QT measurement

For each study, certified technicians digitally recorded resting, supine (or semi-recumbent), standard 12-lead ECGs at study baseline for each participant using Marquette MAC PC machines (GE Healthcare, Milwaukee, WI, USA). The ARIC and WHI CT studies used comparable procedures for preparing participants, placing electrodes, recording, transmitting, processing, and controlling quality of the ECGs. QT was measured electronically using the Marquette 12SL algorithm.

### The Metabochip

The Metabochip was a custom Illumina iSELECT array that contained approximately 195,000 SNPs and was designed to support large scale follow up of putative associations for cardiovascular and metabolic traits including QT, blood pressure, cholesterol, type 2 diabetes, and anthropometrics. Approximately 33% of the Metabochip SNPs were included as replication targets and 62% for fine-mapping. In total, 257 loci were selected for fine-mapping, with the surrounding regions totaling 45.5 Mb accounting for overlaps (14.2 Mb for the densest fine-mapping regions). Eleven QT loci identified in previous GWA studies in populations of European and Asian ancestry were represented on the Metabochip (Table 1). The only published QT locus that is not represented on the Metabochip is an intergenic region on 13q14 reported by Marroni et al [19], but not replicated by other published GWA studies of populations with similar ancestral backgrounds. SNPs reported in the literature but not genotyped on the Metabochip (*NOS1AP*, rs10494366; *NDRG4*, rs7188697, rs37062) were represented by proxies, defined as SNPs in high LD (r^2^≥0.90) with the index SNP using HapMap YRI data.

### Genotyping and quality control assessment

Samples were genotyped at the Human Genetics Center of the University of Texas-Houston and the Translational Genomics Research Institute for ARIC and WHI, respectively, following each genotyping center's standard procedures. HapMap YRI (Yoruba in Ibadan, Nigeria) samples were also genotyped independently by each study to facilitate cross-study quality control. Genotypes were called separately for each study, albeit with a common protocol and common personnel, with GenomeStudio using the GenCall 2.0 algorithm. Because the Metabochip includes SNPs with much lower MAFs than are usually called with GenCall, SNPs were recalled using the GenoSNP genotype-calling algorithm [37]. SNPs with call rates <95%, Hardy-Weinberg equilibrium *P*<10^−6^, >1 Mendelian error (in 30 YRI trios), >2 replication errors, or >3.3% discordant calls in YRI across genotyping centers or against the HapMap database were considered quality control failures. Samples with call rates <0.95 or an inbreeding coefficient F>0.15 were excluded from further analysis [38].

Prior to analyses, related participants were identified using PLINK [39] by estimating identical-by-descent statistics for all pairs. When apparent first-degree relative pairs were identified, the member from each pair with the lower call rate was excluded from further analysis. Principal components of ancestry were determined using the Eigensoft software [40], [41] and apparent ancestral outliers were excluded from further analysis.

### WHI SHARe imputation

Briefly, n = 1,962 WHI participants who were genotyped on both the Affymetrix 6.0 and Metabochip genotyping platforms were used to infer Metabochip genotypes to the n = 8,421 population of WHI participants genotyped on the Affymetrix 6.0 array [36]. Before phasing and imputation, Affymetrix 6.0 SNPs with genotype call rates <90%, Hardy-Weinberg *P*-values<10^−6^, or MAF<0.01 were removed. Participants with call rates <95%, those who demonstrated excess heterozygosity, were part of a first-degree relative pair, or who were identified as an ancestry outlier were excluded. This yielded a set of 987,749 SNPs for the 1,962 reference participants. Mean concordance rates for the 23,703 SNPs in common was 99.7%. Haplotypes were reconstructed using MaCH and were used as a reference to impute Metabochip data into the 6,459 WHI participants with only Affymetrix 6.0 data. Liu et al., (2012) demonstrated the ability to impute 99.9% (97.5%, 83.6%, 52.0%, 20.5%) of SNPs with MAF≥0.05 (0.03–0.05, 0.01–0.03, 0.005–0.01, and 0.001–0.005) with average dosage r^2^ = 94.7% (92.1%, 89.0%, 83.1%, and 79.7%), respectively. For this analysis, all imputed SNPs with r^2^<0.95 were excluded.

### Statistical analysis

To interpret fine-mapping results, LD in our African American PAGE Metabochip sample was calculated in 500 Kb sliding windows using PLINK. In addition, Metabochip LD and frequency information (but not individual-level information) was provided by the Malmö Diet and Cancer Study on 2,143 control participants from a Swedish population [42] to facilitate LD and MAF comparisons to populations of European ancestry. HapMap CEU LD data were used for previously published GWA studies in European populations, as not all European index variants were represented on the Metabochip. Regional association plots use positions from NCBI build 36. Recombination rates were estimated from HapMap phase II data.

Linear regression models were used to study the association between QT and 6,670 SNPs from 11 regions fine-mapped for QT assuming an additive genetic model and including age, sex, study center, ancestry principal components, and heart rate as covariates. Study-specific association results were combined using an inverse variance meta-analysis approach as implemented in METAL [43].

For each QT locus, it is expected that SNPs associated with QT in African Americans will be correlated with the index SNP reported in Europeans. Therefore, we first identified and tested SNPs that are correlated (r^2^≥0.20) with the index signals in Europeans using LD statistics estimated in the Malmö Diet and Cancer Study. In order to determine the appropriate multiple testing threshold for declaration of whether the previously identified signals were significantly associated with QT in African Americans, i.e. generalizability, we then estimated the number of tag SNPs needed to capture all common alleles (r^2^≥0.80) using African American LD patterns. The multiple testing threshold for declaring generalization was α_a_ = 0.05/415, where 415 = the total number of tags identified using African American LD patterns.

To identify significant population-specific SNPs influencing QT that were not correlated with the index signal in Europeans (i.e. r^2^<0.20, which was estimated in the Malmö Diet and Cancer Study), we used an efficient Monte Carlo approach that accounts for LD between SNPs at the previously identified QT loci (α_b_ = 1.37×10^−5^) [44]. Conditional analyses were then performed to determine the number of independent signals the population-specific SNPs represent. Specifically, analyses were repeated for each locus including the SNP with the smallest *P* – value as a covariate. This approach was performed adjusting for successively less significant SNPs until no SNPs with *P* –values lower than α_b_ = 1.37×10^−5^ were identified. To facilitate comparability with previous reports examining the proportion of variance in QT explained by common SNPs, heart rate-corrected QT [45] was regressed on the six best markers in African Americans and the three population-specific variants assuming an additive genetic model and including age, sex, study center, ancestry principal components as covariates.

### Functional categorization of QT loci

For each of the nine QT SNPs (i.e. the six best markers in African Americans and the three novel SNPs), we identified all SNPs in LD (r^2^≥0.5) using the genotypes from the African American population described in this study. We refer to these SNP sets as Trait Associated SNP (TAS) blocks. We assigned each TAS to one or more of the functional annotation datasets listed in Table S5. These datasets are not mutually exclusive. For example, a TAS can reside in both a candidate regulatory element (dataset #7) and a CTCF binding site (dataset #10). For TASs that occur within predicted transcription factor binding sites (datasets #3 and #8), we calculated transcription factor binding affinity for each TAS allele using PWM-scan [46], as described previously [47]. For TASs that occur within 3′ untranslated regions, we used the TargetScanS algorithm to determine whether they disrupt likely microRNA target sites (dataset #5). To define candidate non-promoter regulatory elements of greatest relevance to QT (dataset #7), we restricted the analysis of DNase I hypersensitive sites (open chromatin loci) to only those present in human cardiomyocytes.

## Supporting Information

Figure S1 **−Log** *P* **plot for common SNPs at the** *ATP1B1* **locus.** *P*-values are estimated in African Americans and are plotted using linkage disequilibrium estimates from African Americans (panel A) and Europeans (panel B). SNPs are represented by *circles*, lines indicate index SNPS previously identified in GWA studies of European and Indian Asian populations, and the *large blue diamond* is the best marker in African Americans. Circle color represents correlation with the best marker in African Americans: *blue* indicates weak correlation and *red* indicates strong correlation. Recombination rate is plotted in the background and annotated genes are shown at the bottom of the plot.
(DOCX)

Figure S2 **−Log** *P* **plot for common SNPs at the** *PLN* **independent signal 1 locus.** *P*-values are estimated in African Americans and are plotted using linkage disequilibrium estimates from African Americans (panel A) and Europeans (panel B). SNPs are represented by *circles*, lines indicate index SNPS previously identified in GWA studies of European and Indian Asian populations, and the *large blue diamond* is the best marker in African Americans. Circle color represents correlation with the best marker in African Americans: *blue* indicates weak correlation and *red* indicates strong correlation. Recombination rate is plotted in the background and annotated genes are shown at the bottom of the plot.
(DOCX)

Figure S3 **−Log** *P* **plot for common SNPs at the** *KCNQ1* **locus.** *P*-values are estimated in African Americans and are plotted using linkage disequilibrium estimates from African Americans (panel A) and Europeans (panel B). SNPs are represented by *circles*, lines indicate index SNPS previously identified in GWA studies of European and Indian Asian populations, and the *large blue diamond* is the best marker in African Americans. Circle color represents correlation with the best marker in African Americans: *blue* indicates weak correlation and *red* indicates strong correlation. Recombination rate is plotted in the background and annotated genes are shown at the bottom of the plot.
(DOCX)

Figure S4 **−Log <~/emph>*P*** plot for common SNPs at the ***NDRG4*** locus.***P*-values are estimated in African Americans and are plotted using linkage disequilibrium estimates from African Americans (panel A) and Europeans (panel B). SNPs are represented by *circles*, lines indicate index SNPS previously identified in GWA studies of European and Indian Asian populations, and the *large blue diamond* is the best marker in African Americans. Circle color represents correlation with the best marker in African Americans: *blue* indicates weak correlation and *red* indicates strong correlation. Recombination rate is plotted in the background and annotated genes are shown at the bottom of the plot.**
(DOCX)

Table S1 **Demographic characteristics of n = 8,644 African American participants from four studies.**
(DOCX)

Table S2 **Characterization of 11 genomic regions previously associated with QT and included on the Illumina Metabochip.**
(DOCX)

Table S3 **Associations with common variants at fifteen previously reported QT loci across eleven chromosomes in n = 8,644 African American participants.**
(DOCX)

Table S4 **Associations with common variants at nine previously reported QT loci that did not generalize to n = 8,644 African American participants.**
(DOCX)

Table S5 **Genomic datasets used for bioinformatic characterization of QT loci.**
(DOCX)

Table S6 **Bioinformatic characterization, predicted function, and correlation with index SNP for nine SNPs associated with QT detected in n = 8,644 African American participants.**
(DOCX)

Table S7 **QT functional candidates for evaluation after bioinformatic analysis using data from n = 8,644 African American participants.**
(DOCX)

Text S1 **Descriptions of participating studies.**
(PDF)
