# Supplementary material for: Fine-Mapping and Initial Characterization of QT Interval Loci in African Americans
Source: PLoS Genet. 2012 Aug 9;8(8):e1002870. doi: 10.1371/journal.pgen.1002870 (PMC3415454; doi:10.1371/journal.pgen.1002870)
Supplement: Figure S1 — −Log P plot for common SNPs at the ATP1B1 locus. P-values are estimated in African Americans and are plotted using linkage disequilibrium estimates from African Americans (panel A) and Europeans (panel B). SNPs are represented by circles, lines indicate index SNPS previously identified in GWA studies of European and Indian Asian populations, and the large blue diamond is the best marker in African Americans. Circle color represents correlation with the best marker in African Americans: blue indicates weak correlation and red indicates strong correlation. Recombination rate is plotted in the background and annotated genes are shown at the bottom of the plot. (DOCX) [file pgen.1002870.s001.docx]

FIGURE S1. –Log *P* plot for common SNPs at the *ATP1B1* locus. *P*-values are estimated in African Americans and are plotted using linkage disequilibrium estimates from African Americans (panel A) and Europeans (panel B).
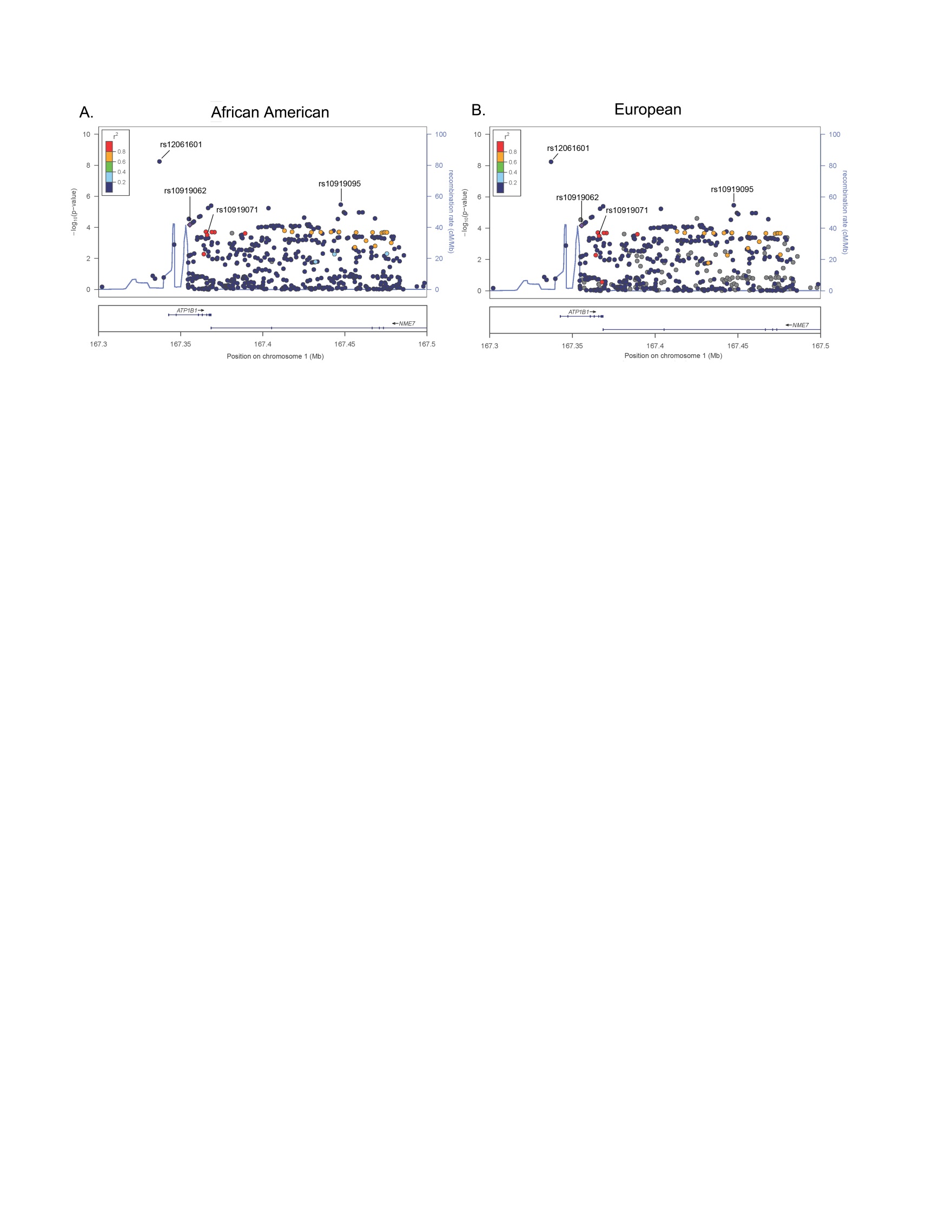


SNPs are represented by *circles*, lines indicate index SNPS previously identified in GWA studies of European and Indian Asian populations, and the *large blue diamond* is the best marker in African Americans. Circle color represents correlation with the best marker in African Americans: *blue* indicates weak correlation and *red* indicates strong correlation. Recombination rate is plotted in the background and annotated genes are shown at the bottom of the plot.
