# Supplementary material for: Fine-Mapping and Initial Characterization of QT Interval Loci in African Americans
Source: PLoS Genet. 2012 Aug 9;8(8):e1002870. doi: 10.1371/journal.pgen.1002870 (PMC3415454; doi:10.1371/journal.pgen.1002870)
Supplement: Figure S2 — −Log P plot for common SNPs at the PLN independent signal 1 locus. P-values are estimated in African Americans and are plotted using linkage disequilibrium estimates from African Americans (panel A) and Europeans (panel B). SNPs are represented by circles, lines indicate index SNPS previously identified in GWA studies of European and Indian Asian populations, and the large blue diamond is the best marker in African Americans. Circle color represents correlation with the best marker in African Americans: blue indicates weak correlation and red indicates strong correlation. Recombination rate is plotted in the background and annotated genes are shown at the bottom of the plot. (DOCX) [file pgen.1002870.s002.docx]

FIGURE S2. –Log *P* plot for common SNPs at the *PLN* independent signal 1 locus. *P*-values are estimated in African Americans and are plotted using linkage disequilibrium estimates from African Americans (panel A) and Europeans (panel B).


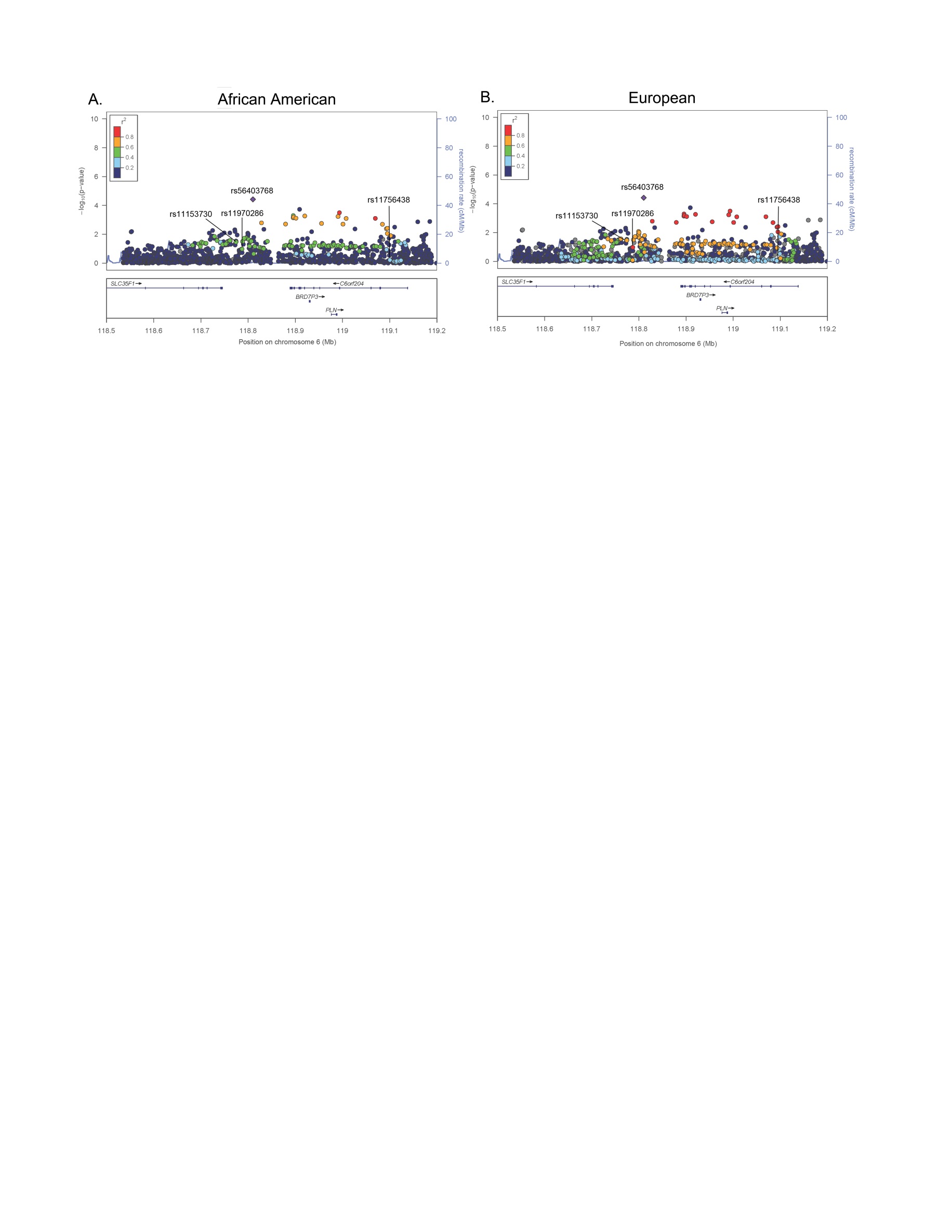


SNPs are represented by *circles*, lines indicate index SNPS previously identified in GWA studies of European and Indian Asian populations, and the *large blue diamond* is the best marker in African Americans. Circle color represents correlation with the best marker in African Americans: *blue* indicates weak correlation and *red* indicates strong correlation. Recombination rate is plotted in the background and annotated genes are shown at the bottom of the plot.
