# Supplementary material for: Fine-Mapping and Initial Characterization of QT Interval Loci in African Americans
Source: PLoS Genet. 2012 Aug 9;8(8):e1002870. doi: 10.1371/journal.pgen.1002870 (PMC3415454; doi:10.1371/journal.pgen.1002870)
Supplement: Table S1 — Demographic characteristics of n = 8,644 African American participants from four studies. (DOCX) [file pgen.1002870.s005.docx]

| **TABLE S1. Demographic characteristics of n=8,644 African American participants from four studies.** | | | | |
| --- | --- | --- | --- | --- |
| **Baseline characteristic** | **ARIC^a^** | **WHI PAGE ^a^** | | **WHI SHARe^b^** |
|  |  | **Wave 1** | **Wave 2** |  |
| N | 3,188 | 797 | 1,128 | 3,531 |
| Age, years, mean (SD) | 54 (6) | 61 (7) | 61 (7) | 62 (7) |
| Sex, female, N (%) | 2,016 (63.2) | 100 | 100 | 100 |
| QT duration, ms, mean (SD) | 402 (31) | 399 (33) | 401 (34) | 401 (34) |
| Heart rate, bpm, mean (SD) | 67 (11) | 68 (11) | 68 (11) | 67 (11) |
| ARIC, Atherosclerosis Risk in Communities Study. Bpm, beats per minute; Ms, milliseconds; PAGE, Population Architecture using Genomics and Epidemiology. SHARe, SNP Health Association Resource. WHI, Women’s Health Initiative. ^a^Directly genotyped on Metabochip.^b^Genotypes imputed from Affymetrix 6.0 panel. | | | | |
