# Supplementary material for: Fine-Mapping and Initial Characterization of QT Interval Loci in African Americans
Source: PLoS Genet. 2012 Aug 9;8(8):e1002870. doi: 10.1371/journal.pgen.1002870 (PMC3415454; doi:10.1371/journal.pgen.1002870)
Supplement: Table S2 — Characterization of 11 genomic regions previously associated with QT and included on the Illumina Metabochip. (DOCX) [file pgen.1002870.s006.docx]

| **TABLE S2. Characterization of 11 genomic regions previously associated with QT and included on the Illumina Metabochip.** | | | | | |
| --- | --- | --- | --- | --- | --- |
| **Previously**  **identified locus** | **Genetic region** | **Base pair range**  **(Build 36)** | **N SNPs**  **fine-mapped^a^** | **Imputation quality** | |
|  |  |  |  | **Median** | **IQR** |
| *RNF207* [[3](#_ENREF_3),[4](#_ENREF_4)] | 1p36.31 | 6180734, 6247856 | 51 | 0.86 | 0.82, 0.99 |
| *NOS1AP* [[3-8](#_ENREF_3)] | 1q23.3 | 160219639, 160705156 | 1,184 | 0.99 | 0.95, 0.99 |
| *ATP1B1* [[4](#_ENREF_4)] | 1q24.2 | 167339674, 167507362 | 441 | 0.99 | 0.99, 0.99 |
| *SCN5A* [[3](#_ENREF_3),[4](#_ENREF_4)] | 3p22.2 | 38490026, 38818967 | 656 | 0.98 | 0.95, 0.99 |
| *PLN, SLC35F1* [[3-5](#_ENREF_3)] | 6q22.31 | 118535289, 119198946 | 1,081 | 0.99 | 0.98, 0.99 |
| *KCNH2* [[3](#_ENREF_3),[4](#_ENREF_4)] | 7q36.1 | 150217630, 150338421 | 265 | 0.76 | 0.68, 0.88 |
| *KCNQ1* [[3](#_ENREF_3),[4](#_ENREF_4)] | 11p15.5 | 2374469, 2902982 | 1,360 | 0.86 | 0.76, 0.94 |
| *LITAF* [[3](#_ENREF_3),[4](#_ENREF_4)] | 16p13.13 | 11574706, 11655762 | 91 | 0.91 | 0.84, 0.97 |
| *NDRG4* [[3](#_ENREF_3),[4](#_ENREF_4)] | 16q21 | 57085476, 57255408 | 307 | 0.98 | 0.89, 0.99 |
| *LIG3,RFFL* [[3](#_ENREF_3)] | 17q12 | 30129498, 30469662 | 382 | 0.99 | 0.97, 0.99 |
| *KCNJ2* [[4](#_ENREF_4)] | 17q24 | 65762031, 66037656 | 852 | 0.99 | 0.98, 0.99 |
| ^a^Restricted to SNPs with minor allele frequency > 0.01. N, number. Kb, kilobase. SNP, single nucleotide polymorphism. | | | | | |
