# Supplementary material for: Fine-Mapping and Initial Characterization of QT Interval Loci in African Americans
Source: PLoS Genet. 2012 Aug 9;8(8):e1002870. doi: 10.1371/journal.pgen.1002870 (PMC3415454; doi:10.1371/journal.pgen.1002870)
Supplement: Table S3 — Associations with common variants at fifteen previously reported QT loci across eleven chromosomes in n = 8,644 African American participants. (DOCX) [file pgen.1002870.s007.docx]

| **TABLE S3. Associations with common variants at fifteen previously reported QT loci across eleven chromosomes in n=8,644 African American participants.** | | | | | | | | | | | | |
| --- | --- | --- | --- | --- | --- | --- | --- | --- | --- | --- | --- | --- |
| **Index SNPs from Published GWA studies in European and Indian Asian populations** | | | | | | | **Best marker in African Americans**^a^ | | | | **D^’^ with index SNP** | |
| **Locus** | **Position** | **Ind. signal** | **Index SNP** | **Alleles** | **CAF** | | **Marker** | **BP**  **(build 36)** | **Alleles** | **CAF** | **EU^b^** | **AF^c^** |
|  |  |  |  |  | **EU^b^** | **AF^c^** |  |  |  |  |  |  |
| *NOS1AP* | 1q23.3 | 1 | rs12143842 [[20-22](#_ENREF_20)] | T/C | 0.24 | 0.13 | rs12143842 | 160300514 | T/C | 0.13 | 1.0 | 1.0 |
|  |  |  | rs2880058 [[19](#_ENREF_19),[23](#_ENREF_23)] | A/G | 0.30 | 0.29 | --- | --- | --- | --- | 0.92 | 1.0 |
|  |  |  | rs10494366^d^ [[18](#_ENREF_18)] | A/G | 0.64 | 0.60 | --- | --- | --- | --- | 0.58 | 0.35 |
|  |  | 2 | rs12029454 [[20](#_ENREF_20)] | A/G | 0.15 | 0.28 | rs72633699 | 160431793 | T/C | 0.30 | 1.0 | 1.0 |
|  |  |  | rs4657178 [[22](#_ENREF_22)] | T/C | 0.27 | 0.36 | --- | --- | --- | --- | 1.0 | 0.19 |
| *ATP1B1* | 1q24.2 | 1 | rs10919071 [[22](#_ENREF_22)] | A/G | 0.89 | 0.97 | rs10919062 | 167355571 | T/C | 0.97 | 1.0 | 1.0 |
| *PLN* | 6q22.31 | 1 | rs11970286 [[22](#_ENREF_22)] | T/C | 0.49 | 0.22 | rs56403768 | 118810227 | T/C | 0.77 | 1.0 | 0.92 |
|  |  |  | rs11153730 [[21](#_ENREF_21)] | T/C | 0.53 | 0.71 | --- | --- | --- | --- | 1.0 | 0.94 |
|  |  |  | rs11756438 [[20](#_ENREF_20)] | A/C | 0.48 | 0.36 | --- | --- | --- | --- | 0.82 | 0.74 |
| *KCNQ1* | 11p15.5 | 1 | rs12296050 [[22](#_ENREF_22)] | T/C | 0.81 | 0.52 | rs12296050 | 2445918 | T/C | 0.52 | 1.0 | 1.0 |
|  |  |  | rs12576239 [[20](#_ENREF_20)] | T/C | 0.13 | 0.18 | --- | --- | --- | --- | 1.0 | 0.76 |
|  |  |  | rs2074238^f^ [[20](#_ENREF_20)] | NA | NA | NA | --- | --- | --- | --- | --- | --- |
| *NDRG4* | 16q21 | 1 | rs7188697^d^ [[22](#_ENREF_22)] | T/C | 0.75 | 0.85 | rs7184114 | 57142870 | A/C | 0.83 | 0.95 | 1.0 |
|  |  |  | rs37062^d^ [[20](#_ENREF_20)] | A/G | 0.24 | 0.16 | --- | --- | --- | --- | 1.0 | 1.0 |
| ^a^Restricted to SNPs with minor allele frequency > 0.01.^b^Calculated in the 1,000 Genomes CEU data. ^c^Calculated in the 1,000 Genomes YRI data or HapMap 3 (release 2) ASW data. ^d^SNP not present on Metabochip, SNP proxy substituted. ^e^SNP not present on Metabochip, but in very high LD with rs2968863 (r^2^ > 0.95). ^f^SNP failed quality control and no proxy was available. AF, African American. BP, base pair. CAF, coded allele frequency. Est, estimate. EU, European. GWA, genome wide association. Ind, independent. NA, not available. SE, standard error. SNP, single nucleotide polymorphism. | | | | | | | | | | | | |
