# Supplementary material for: Fine-Mapping and Initial Characterization of QT Interval Loci in African Americans
Source: PLoS Genet. 2012 Aug 9;8(8):e1002870. doi: 10.1371/journal.pgen.1002870 (PMC3415454; doi:10.1371/journal.pgen.1002870)
Supplement: Table S4 — Associations with common variants at nine previously reported QT loci that did not generalize to n = 8,644 African American participants. (DOCX) [file pgen.1002870.s008.docx]

| **TABLE S4. Associations with common variants at nine previously reported QT loci that did not generalize to n=8,644 African American participants.** | | | | | | | | | | | |
| --- | --- | --- | --- | --- | --- | --- | --- | --- | --- | --- | --- |
| **Index SNPs from Published GWA studies in European populations** | | | | | | | | **African Americans** | | | |
| **Locus** | **Position** | **Ind. signal** | **Index SNP** | **Alleles** | **CAF** | **Est.** | **SE** | **CAF^a^** | **Est.** | **SE** | ***P*-value** |
| *RNF207* | 1p36.31 | 1 | rs846111 [[3](#_ENREF_3)] | C/G | 0.29 | 1.49 | 0.25 | 0.056 | 1.15 | 0.94 | 0.22 |
|  |  |  | rs846111 [[4](#_ENREF_4)] | C/G | 0.29 | 2.14 | 0.36 | --- | --- | --- | --- |
| *NOS1AP* | 1q23.3 | 3 | rs16857031 [[3](#_ENREF_3)] | G/C | 0.14 | 3.32 | 0.35 | 0.29 | 0.95 | 0.35 | 7.2 x 10^-3^ |
| *SCN5A* | 3p22.2 | 1 | rs12053903 [[3](#_ENREF_3)] | C/T | 0.34 | -1.40 | 0.175 | 0.81 | 0.16 | 0.40 | 0.70 |
|  |  |  | rs11129795 [[4](#_ENREF_4)] | A/G | 0.23 | -1.27 | 0.23 | 0.18 | -0.52 | 0.41 | 0.20 |
| *PLN* | 6q22.31 | 2 | rs12210810 [[4](#_ENREF_4)] | C/G | 0.06 | -3.13 | 0.43 | 0.010 | -0.67 | 1.75 | 0.70 |
| *KCNH2* | 7q36.1 | 1 | rs2968863 [[4](#_ENREF_4)] | T/C | 0.29 | -1.35 | 0.23 | 0.053 | -1.31 | 0.74 | 0.074 |
|  |  |  | rs2968864^b^ [[3](#_ENREF_3)] | NA | NA | NA | NA | NA | NA | NA | NA |
|  |  | 2 | rs4725982 [[3](#_ENREF_3)] | T/C | 0.22 | 1.575 | 0.175 | 0.26 | 1.25 | 0.49 | 0.010 |
| *LITAF* | 16p13.13 | 1 | rs8049607 [[3](#_ENREF_3)] | T/C | 0.49 | 1.456 | 0.263 | 0.45 | 0.82 | 0.43 | 0.057 |
|  |  |  | rs8049607 [[4](#_ENREF_4)] | T/C | 0.46 | 1.24 | 0.22 | --- | --- | --- | --- |
| *LIG3* | 17q12 | 1 | rs2074518 [[3](#_ENREF_3)] | T/C | 0.46 | -1.23 | 0.175 | 0.22 | -0.65 | 0.43 | 0.13 |
| *KCNJ2* | 17q24 | 1 | rs17779747 [[4](#_ENREF_4)] | T/G | 0.35 | -1.16 | 0.21 | 0.097 | -0.27 | 0.56 | 0.62 |
| ^a^Calculated in the Atherosclerosis Risk in Communities Study. ^b^SNP not present on Metabochip, but in very high LD with rs2968863 (r^2^ > 0.95). CAF, coded allele frequency. Est, estimate. Ind, independent. NA, not available. SE, standard error. SNP, single nucleotide polymorphism. | | | | | | | | | | | |
