# Supplementary material for: Fine-Mapping and Initial Characterization of QT Interval Loci in African Americans
Source: PLoS Genet. 2012 Aug 9;8(8):e1002870. doi: 10.1371/journal.pgen.1002870 (PMC3415454; doi:10.1371/journal.pgen.1002870)
Supplement: Table S5 — Genomic datasets used for bioinformatic characterization of QT loci. (DOCX) [file pgen.1002870.s009.docx]

| **TABLE S5. Genomic datasets used for bioinformatic characterization of QT loci.** | | | |
| --- | --- | --- | --- |
| **Dataset** | **Genomic class** | **Description** | **Data source/program** |
| 1 | Non-synonymous coding | Exonic positions wherein the variant would cause an amino acid replacement | dbSNP version 131 |
| 2 | Promoter | 1kb regions upstream of annotated transcription start sites | RefSeq |
| 3 | Promoter TFBS | Transcription factor binding sites (TFBS) predicted in promoter regions | PWM-scan^a^ |
| 4 | Non-coding RNA | All types of experimentally supported non-coding RNA, including microRNAs | RNAdb 2.0 &  miRBase 17.0 |
| 5 | MicroRNA target site | Computationally predicted microRNA target sites within 3’ UTRs | TargetScanS 5.2 |
| 6 | Enhancer element | Experimentally supported enhancer elements in the heart | VISTA Enhancer Browser |
| 7 | Candidate non-promoter regulatory element | Non-promoter open chromatin loci in human cardiomyocytes (HCM), as assessed by DNase I hypersensitivity (DHS) mapping | UCSC Table Browser: UW DNase I HS data from HCM cells |
| 8 | Candidate non-promoter regulatory element TFBS | Transcription factor binding sites (TFBS) predicted in candidate non-promoter regulatory elements | PWM-scan^a^ |
| 9 | Predicted transcriptional regulatory module | Computationally predicted *cis*-regulatory modules | PReMod database |
| 10 | Insulator elements | CTCF binding sites assessed by ChIP-chip technology | UCSC Table Browser: ORegAnno |
| 11 | MCS elements | Most Conserved Sequences across 17 vertebrates | UCSC Table Browser:  17-way most conserved |
| ^a^PWM-scan was applied using positional weight matrices (PWMs) from the Transfac database. | | | |
