# Supplementary material for: Fine-Mapping and Initial Characterization of QT Interval Loci in African Americans
Source: PLoS Genet. 2012 Aug 9;8(8):e1002870. doi: 10.1371/journal.pgen.1002870 (PMC3415454; doi:10.1371/journal.pgen.1002870)
Supplement: Table S6 — Bioinformatic characterization, predicted function, and correlation with index SNP for nine SNPs associated with QT detected in n = 8,644 African American participants. (DOCX) [file pgen.1002870.s010.docx]

| **TABLE S6. Bioinformatic characterization, predicted function, and correlation with index SNP for nine SNPs associated with QT detected in n=8,644 African American participants.** | | | | | | | | |
| --- | --- | --- | --- | --- | --- | --- | --- | --- |
| **Locus** | **Best marker in African Americans** | **Synonymous site**^a^ | **Non-coding MCS**^a^ | **Promoter**^a^ | **Promoter TFBS**^a,^**^b^** | **Transcriptional regulatory module**^a,^**^b^** | **MicroRNA target site**^a,^**^b^** | **HCM DHS**^a^ |
| *NOS1AP* | rs12143842^c^ | --- | --- | --- | --- | --- | --- | --- |
|  | rs79163067 | --- | --- | --- | --- | --- | --- | --- |
|  | rs72633699 | --- | --- | --- | --- | --- | --- | --- |
| *ATP1B1* | rs10919062 | --- | --- | --- | --- | --- | --- | --- |
|  | rs10919095 | --- | rs909931 (0.75) | --- | --- | --- | --- | --- |
|  | rs12061601^d^ | --- | --- | --- | --- | --- | --- | --- |
| *PLN* | rs56403768 | --- | rs6928210 (0.56) | --- | --- | --- | --- | rs763254 (0.84) |
| *KCNQ1* | rs12296050 | --- | --- | rs3864884 (0.85) | rs3864884 (0.85) | --- | --- | --- |
| *NDRG4* | rs7184114 | rs3743567 (0.54); rs246258 (0.90) | rs3743567 (0.54); rs246258 (0.90) | rs1646010 (0.90); rs27097 (0.89) | rs1646010 (0.90); rs27097 (0.89) | --- | rs37036 (0.91) | --- |
| ^a^Presented as SNP (r^2^); ^b^Predicted; ^c^1,164 bps from closest HCM DHS, < 5kb from annotated *NOS1AP* promoter; ^d^1,147 bps from closest HCM DHS, < 6kb from annotated *NOS1AP* promoter; DHS, DNase hypersensitive site; HCM, human cardiomyocytes; Kb, kilobase. MCS, most conserved sequences; TFBS, transcription factor binding sites | | | | | | | | |
