# Supplementary material for: Fine-Mapping and Initial Characterization of QT Interval Loci in African Americans
Source: PLoS Genet. 2012 Aug 9;8(8):e1002870. doi: 10.1371/journal.pgen.1002870 (PMC3415454; doi:10.1371/journal.pgen.1002870)
Supplement: Table S7 — QT functional candidates for evaluation after bioinformatic analysis using data from n = 8,644 African American participants. (DOCX) [file pgen.1002870.s011.docx]

| **TABLE S7. QT functional candidates for evaluation after bioinformatic analysis using data from n=8,644 African American participants.** | | | | | |
| --- | --- | --- | --- | --- | --- |
| **Locus** | **Best marker in African Americans** | **SNP in LD** | **Annotation** | **Reference allele only** | **Non-reference allele only** |
| *KCNQ1* | rs12296050 | rs3864884 | *KCNQ1* promoter | HAIRY, PAX-3, AP-2 | AHR, TBX5 |
| *NDRG4* | rs7184114 | rs1646010 | *SETD6* promoter | NRSF | --- |
|  |  | rs27097 | *SETD6* promoter | --- | HOXA5 |
|  |  | rs37036 | *SETD6* 3' UTR | miR-624 | --- |
| AHR, aryl hydrocarbon receptor; AP-2, activator protein 2; HAIRY, hairy related transcription factors; HOXA5, homeobox type A5; LD, linkage disequilibrium. miR-624, microRNA-624. NRSF, neural restrictive silencer factor; PAX-3, paired box 3; TBX5, T-box transcription factor 5; UTR, untranscribed region. | | | | | |
