# Supplementary material for: Fine-Mapping and Initial Characterization of QT Interval Loci in African Americans
Source: PLoS Genet. 2012 Aug 9;8(8):e1002870. doi: 10.1371/journal.pgen.1002870 (PMC3415454; doi:10.1371/journal.pgen.1002870)
Supplement: Text S1 — Descriptions of participating studies. (PDF) [file pgen.1002870.s012.pdf]

Atherosclerosis Risk in Communities Study (ARIC)

The ARIC study is an ongoing population-based cohort of 15,792 males and females aged 45-64 years at baseline selected using probability sampling from four United States communities (Forsyth County NC, Jackson MS, suburban Minneapolis MN, and Washington County MD) [1]. Participants, who were predominantly of Caucasian and African-American descent, were recruited in 1987-1989 to examine cardiovascular and pulmonary disease, patterns of medical care, and disease variation over time. Standardized physical examinations and interviewer-administered questionnaires were conducted at baseline, and at three triennial follow-up examinations. The Institutional Review Board at each participating institution approved the ARIC study and all participants provided informed consent before each examination.

Women's Health Initiative Clinical Trial (WHI CT)

The WHI comprises both randomized clinical trials (CT) and an observational study (OS). This study is limited to WHI CT participants, as ECGs were not available for WHI OS participants. The three WHI clinical trials were designed to allow randomized, controlled evaluation of 1) estrogen with or without progestin treatment, 2) calcium/vitamin D supplementation, and 3) dietary modification on the risk of breast and colorectal cancer, cardiovascular disease, and bone fractures [2]. Between 1993 and 1998, the trials enrolled 68,132 postmenopausal women aged 50–79 years who were followed at 1 of 75 US examination sites (including satellites, remote sites, and their changes in location). Women were ineligible if they had medical conditions predictive of survival time less than 3 years, if they were known to have conditions inconsistent with

study participation and adherence, or if they were active participants in another randomized, controlled trial. Those who remained eligible and interested were invited to follow-up examinations at 1, 3, 6, and 9 years. The Institutional Review Board at each participating institution approved the WHI CT study and all participants provided informed consent before each examination.

Supplemental References

1. ARIC Investigators (1989) The Atherosclerosis Risk in Communities (ARIC) Study: design and objectives. The ARIC investigators. Am J Epidemiol 129: 687-702.

2. WHI Study Group (1998) Design of the Women's Health Initiative clinical trial and observational study. The Women's Health Initiative Study Group. Control Clin Trials 19: 61-109.
